# A Major QTL Located in Chromosome 8 of *Cucurbita moschata* Is Responsible for Resistance to *Tomato Leaf Curl New Delhi Virus*

**DOI:** 10.3389/fpls.2020.00207

**Published:** 2020-03-20

**Authors:** Cristina Sáez, Cecilia Martínez, Javier Montero-Pau, Cristina Esteras, Alicia Sifres, José Blanca, María Ferriol, Carmelo López, Belén Picó

**Affiliations:** ^1^Institute for the Conservation and Breeding of Agricultural Biodiversity, Universitat Politècnica de València, Valencia, Spain; ^2^Agrifood Campus of International Excellence (ceiA3), Department of Biology and Geology, Universidad de Almería, Almería, Spain; ^3^Department of Biochemistry and Molecular Biology, Universitat de València, Valencia, Spain; ^4^Instituto Agroforestal Mediterráneo, Universitat Politècnica de València, Valencia, Spain

**Keywords:** ToLCNDV, resistance, *Cucurbita*, zucchini, QTL, synteny

## Abstract

*Tomato leaf curl New Delhi virus* (ToLCNDV) is a bipartite whitefly transmitted begomovirus, responsible since 2013 of severe damages in cucurbit crops in Southeastern Spain. Zucchini (*Cucurbita pepo*) is the most affected species, but melon (*Cucumis melo*) and cucumber (*Cucumis sativus*) are also highly damaged by the infection. The virus has spread across Mediterranean basin and European countries, and integrated control measures are not being enough to reduce economic losses. The identification of resistance genes is required to develop resistant cultivars. In this assay, we studied the inheritance of the resistance to ToLCNDV previously identified in two *Cucurbita moschata* accessions. We generated segregating populations crossing both resistant pumpkins, an American improved cultivar Large Cheese (PI 604506) and an Indian landrace (PI 381814), with a susceptible *C. moschata* genotype (PI 419083). The analysis of symptoms and viral titers of all populations established the same monogenic recessive genetic control in both resistant accessions, and the allelism tests suggest the occurrence of alleles of the same *locus*. By genotyping with a single nucleotide polymorphism (SNP) collection evenly distributed along the *C*. *moschata* genome, a major quantitative trait locus (QTL) was identified in chromosome 8 controlling resistance to ToLCNDV. This major QTL was also confirmed in the interspecific *C. moschata* × *C. pepo* segregating populations, although *C. pepo* genetic background affected the resistance level. Molecular markers here identified, linked to the ToLCNDV resistance *locus*, are highly valuable for zucchini breeding programs, allowing the selection of improved commercial materials. The duplication of the candidate region within the *C. moschata* genome was studied, and genes with paralogs or single-copy genes were identified. Its synteny with the region of chromosome 17 of the susceptible *C. pepo* revealed an INDEL including interesting candidate genes. The chromosome 8 candidate region of *C. moschata* was also syntenic to the region in chromosome 11 of melon, previously described as responsible of ToLCNDV resistance. Common genes in the candidate regions of both cucurbits, with high- or moderate-impact polymorphic SNPs between resistant and susceptible *C. moschata* accessions, are interesting to study the mechanisms involved in this recessive resistance.

## Introduction

*Tomato leaf curl New Delhi virus* (ToLCNDV) is an economically important begomoviru*s* (family *Geminiviridae*) with two circular single-stranded DNA genome components of ∼2.7 kb, designated as DNA-A and DNA-B (Padidam et al., 1995; Jyothsna et al., 2013). ToLCNDV is transmitted in nature by the whitefly *Bemisia tabaci* biotypes MEAM1 and MED (Chang et al., 2010; Rosen et al., 2015; Janssen et al., 2017), but some isolates of this virus can also be transmitted by mechanical inoculation (Usharani et al., 2004; Chang et al., 2010; Sohrab et al., 2013; López et al., 2015).

ToLCNDV has a wide host range. It affects crops of the *Solanaceae* family, such as tomato (*Solanum lycopersicum* L.), potato (*Solanum tuberosum* L.), chili pepper (*Capsicum annuum* L.), and eggplant (*Solanum melongena* L.) (Padidam et al., 1995; Hussain et al., 2004; Usharani et al., 2004; Pratap et al., 2011). It is also highly damaging to crops of the *Cucurbitaceae* family, including luffa (*Luffa cylindrica* M. Roem.), ash gourd [*Benincasa hispida* (Thunb.) Cogn.], cucumber (*Cucumis sativus* L.), watermelon (*Citrullus lanatus* L), melon (*C. melo* L.), and different types of squashes (*Cucurbita* spp.) (Sohrab et al., 2003; Ito et al., 2008; Singh et al., 2009; Chang et al., 2010; Roy et al., 2013). Recently, it has been reported to be affecting species of other plant families, such as opium poppy (*Papaver somniferum* L., Papaveraceae) (Srivastava et al., 2016), cotton (*Gossypium hirsutum* L., Malvaceae) (Zaidi et al., 2016), soybean (*Glycine max*, Fabaceae L. Merr.) (Jamil et al., 2017), and firecracker flower (*Crossandra infundibuliformis* L. Nees, *Acanthaceae*) (Sundararaj et al., 2019). Furthermore, some weeds as black nightshade (*Solanum nigrum* L.), thorn apple (*Datura stramonium* L.), squirting cucumber [*Ecballium elaterium* (L.) A. Rich], smooth sowthistle (*Sonchus oleraceus* L.), false daisy [*Eclipta prostrata* (L.) L.], and apple of Sodom [*Calotropis procera* (Aiton) Dryand.] (Haider et al., 2006; Moriones et al., 2017; Zaidi et al., 2017; Juárez et al., 2019) have been found to be hosts of the virus, acting as reservoirs during the whole year.

ToLCNDV was first detected in North India in 1995 (Srivastava et al., 1995), from where it spread to South and Southeast Asian countries. It was limited to Asia until 2012, when it was reported affecting cucurbits in different Mediterranean countries, first in Spain (Juárez et al., 2014) and later in Tunisia (Mnari-Hattab et al., 2015), Italy (Panno et al., 2016), Morocco (Sifres et al., 2018), Greece (Orfanidou et al., 2019), and Algeria (Kheireddine et al., 2019). More recently, the virus has been identified in cucurbits plants in Portugal and Estonia (EPPO, 2019), which is indicative of the rapid spread of ToLCNDV through Europe. The most affected crop in European countries is Zucchini squash (*Cucurbita pepo* L. subsp. *pepo*). In this crop, the virus causes severe stunting of plants, which exhibit upward and downward curling of the leaves, severe mosaic, and fruit skin roughness (Juárez et al., 2014). Infected plants often present partial or complete yield loss and fruits with lower market value. Zucchini is one of the most widely grown crops and appreciated vegetable in the Mediterranean basin. This region produced nearly 300,000 tm of this vegetable and other species of the *Cucurbita* genus (pumpkins, squash, and gourds) in FAO (2017), representing almost 24% of world production, excluding China and India. Before the arrival of ToLCNDV, the aphid-borne potyvirus *Zucchini yellow mosaic virus* (ZYMV) was the major viral pathogen of this crop (Capuozzo et al., 2017). Since 2013, ToLCNDV is the most prevalent virus in the area, where it is an important constraint to zucchini production. In the background of the severe epidemic outbreaks of ToLCNDV in cucurbits, both in greenhouses and in open fields, European and Mediterranean Plant Protection Organization (EPPO) has added this virus to the EPPO Alert List (EPPO, 2017).

Cultural practices, such as the control of the whitefly vector, the elimination of infected plants, and the avoidance of the most susceptible cultivars, are not very effective in preventing ToLCNDV outbreaks (EPPO, 2017). In fact, breeding resistant varieties is considered the most economical and effective method to control virus diseases. Genetic resistance to ToLCNDV has been identified in some accessions of the *Cucurbita* genus (Sáez et al., 2016). In that work, authors screened for ToLCNDV resistance in a large collection of *Cucurbita* spp. accessions including landraces and commercial varieties of the cultivated species (*C. pepo* L., *C. moschata* Duchesne and *C. maxima* Duchesne) and wild *Cucurbita* species. All the *C. pepo* and *C. maxima* accessions behaved as highly susceptible, but four *C. moschata* accessions were highly resistant, two of them after both mechanical and whitefly inoculation, remaining symptomless with a reduced viral accumulation (Sáez et al., 2016).

Genetic resistance to ToLCNDV has also been characterized in some other species belonging to different families. In *Solanum habrochaites* S. Knapp & D.M. Spooner, a wild species related to tomato, three dominant genes are responsible for the resistance (Rai et al., 2013). In *L. cylindrica*, a popular cucurbit vegetable in India, a dominant monogenic resistance was reported (Islam et al., 2010, 2011). More recently, in melon, Sáez et al. (2017) found one major *locus* in chromosome 11 and two additional regions in chromosomes 12 and 2 that control resistance to ToLCNDV. In this context, the purpose of this study was to map the quantitative trait loci (QTL) associated with the resistance to ToLCNDV in *C. moschata* using segregating populations derived from these resistant sources and a susceptible accession of this species, and to confirm this resistance in interspecific *C. moschata* × *C. pepo* populations as the first step to transfer the resistance to zucchini.

## Materials and Methods

### Plant Material

In this work, we selected two *Cucurbita moschata* accessions (PI 604506 and PI 381814), previously reported (Sáez et al., 2016) as symptomless or with slight symptoms after whitefly and sap inoculation with ToLCNDV, to study the genetic control of the resistance. PI 604506 is the improved pumpkin cultivar Large Cheese from the United States and PI 381814, an Indian landrace. The Chinese *C. moschata* accession PI 419083 was used as susceptible control. Seeds of the three accessions were first provided by the United States Department of Agriculture-National Plant Germplasm System (USDA-NPGS) genebank, then fixed by selfing and multiplied by the cucurbits breeding group at the Institute for the Conservation and Breeding of Agricultural Biodiversity (COMAV), and stored at the COMAV genebank.

### Virus Source and Mechanical Inoculation

To generate the viral inoculum source, susceptible zucchini plants were agroinfiltrated with an infectious clone based on the Spanish isolate of ToLCNDV (99% nucleotide identity with the sequences of the A and B viral genomic particles: KF749224 and KF749225 (Juárez et al., 2014), following the procedure described in Sáez et al. (2016).

The tissue of symptomatic leaves from 15 days post-ToLCNDV agroinoculation plants was crushed in a mortar together with inoculation buffer [50 mM potassium phosphate (pH 8.0), 1% polyvinylpyrrolidone 10, 1% polyethylene glycol 6000, 10 mM 2-mercaptoethanol and 1% activated charcoal] in a 1:4 (*w*/*v*) proportion (López et al., 2015). The homogenate was used to mechanically inoculate all plants at the stage of one true leaf, dusting on the true leaf and on one cotyledon with Carborundum 600 mesh and scratching with a cotton swab dipped in the blend. Inoculated plants were grown in a climatic chamber, and disease progression was monitored. Symptomless plants 15 days after mechanical inoculation (dpi) were reinoculated to avoid escaping to the infection.

### Generation of F_1_ and Segregating Populations

Ten seeds of each *C. moschata* accession were disinfected and germinated as described by Sáez et al. (2016). Seedlings were transplanted to pots and grown in climatic chamber under controlled conditions (photoperiod of 16 h day at 25°C and 8 h night at 18°C and 70% of relative humidity). Subsequently, plants were moved to a greenhouse and crossed to obtain three F_1_ progenies: F_1_ PI 419083 × PI 604506, F_1_ PI 419083 × PI 381814, and F_1_ PI 604506 × PI 381814. Eight plants of each parent and the corresponding hybrids were mechanically inoculated with ToLCNDV as described above and phenotyped according to symptomatology and viral accumulation as described below.

Eight additional plants of the *C. moschata* parents were cultivated in a greenhouse along with eight plants of the F_1_ progenies. To generate segregating populations, F_1_ plants were selfed to obtain F_2_ progenies and backcrossed to plants of PI 604506, PI 381814, and PI 419083 to generate the BC1_PI 604506_, BC1_PI 381814_, and BC1_PI 419083_ populations, respectively. All these segregating populations were screened against ToLCNDV with the same inoculation and phenotyping methodology, using three plants of each *C. moschata* accession as controls. F_2_ and BC1 derived from F_1_ PI 419083 × PI 381814 were obtained later because of the influence of the local climate conditions in PI 381814 vegetative growth, causing late-flowering and slow development of fruits. Hence, we studied first the genetic control of the resistance to ToLCNDV in the segregating populations derived from PI 604506, and results were validated in F_2_ and BC1 coming from F_1_ PI 419083 × PI 381814.

### Symptoms Evaluation and Quantification of the Viral Accumulation

Symptomatology was evaluated in all plants at 15 and 30 dpi using the visual scale described by López et al. (2015). Symptoms score ranged from 0 (absence of symptoms) to 4 (highly severe symptoms), classifying as resistant those plants with symptoms scored 0 or 1 and as susceptible those with symptoms scored from 2 to 4. The goodness-of-fit between the expected and observed segregation ratios resistant/susceptible plants was analyzed by chi-squared (χ^2^) test (*p* < 0.05) in the F_2_ and BC1-segregating populations.

The relative ToLCNDV accumulation in each plant was determined at 30 dpi by quantitative PCR (qPCR). Total DNA from apical leaves was extracted using the cetyltrimethyl ammonium bromide (CTAB) method (Doyle and Doyle, 1990) and quantified using a NanoDrop 1000 spectrophotometer (Thermo Scientific, Waltham, MA, United States). DNA was diluted with sterile-deionized water to a final concentration of 5 ng μl^–1^. Three biological replicates were done for each parental genotype, and all plants of the assay were analyzed in three technical replicates using a *LightCycler*^®^ 480 System (Roche). In each qPCR reaction, 15 ng of genomic DNA were used as templates, in a final volume of 15 μl. We used 7.5 μl of 2 × iTaq^TM^ universal SYBR^®^ Green Supermix (BIO-RAD) and 1.5 μl (100 nM) of each primer and 1.5 μl of H_2_O. Primers ToLCNDVF1 (5′-AATGCCGACT ACACCAAGCAT-3′, positions 1145–1169) and ToLCNDVR1 (5′-GGATCGAGCAGAGAGTGGCG-3′, positions 1399–1418) were used for the amplification of a 273-bp fragment of viral DNA-A. The single-copy gene *CpACS2* was amplified in all samples as internal control using the primers CpACS2F (5′-ACT CGATCAACTTCGAGCAAA-3′) and CpACS2R (5′-GCCTA TCCAAAGACCTCGGCCTTCCC-3′). Both ToLCNDVF1/R1 and *CpACS2* primers were used in previous works by Sáez et al. (2016). Cycling conditions consisted of incubation at 95°C for 5 min, 45 cycles of 95°C for 5 s and 60°C for 30 s. Relative ToLCNDV levels were calculated using the 2^–ΔCt^ expression, a variation of the Livak method (Livak and Schmittgen, 2001; Bio-Rad Laboratories, 2006), where ratio (target/reference) = 2^–ΔCt^ = 2^–[Ct (viral target)^^–^^Ct (reference gen)]^.

### QTL Analysis in *C. moschata* F_2_ Population Derived From PI 419083 × PI 604506

PI 604506 and PI 419083 accessions were included in an RNAseq analysis, performed in the frame of a *de novo* assembly of the zucchini genome project (Montero-Pau et al., 2018), and their transcriptome sequences were used to generate the single nucleotide polymorphism (SNP) panel used here. SNPs were selected by aligning each sequence to the version 1 of the *C. moschata* cv. *Rifu* genome (Sun et al., 2017), available at the Cucurbit Genomics Database^1^. We used the Bowtie2 tool with the very-sensitive-local argument. Variant calling was performed using Freebayes version 1.0.2 (Garrison and Marth, 2012), excluding alignments from the analysis if they had a mapping quality < 40, alleles with quality under 20, and filtering SNPs with minimum count of 10. A set of 137 SNPs evenly distributed throughout the *C. moschata* genome (Supplementary Table 1) were selected and used to genotype PI 604506, PI 419083, their derived F_1_, and 134 plants of the corresponding F_2_ population.

All plants were genotyped using the Agena Bioscience iPLEX^®^ Gold MassARRAY (Agena Biosciences) system at the Epigenetic and Genotyping unit of the University of Valencia [Unitat Central d’Investigació en Medicina (UCIM), Faculty of Medicine, Spain]. Total DNA was extracted from the tissue of young leaves, using the protocol described above, and quantified and adjusted to 15 ng μl^–1^. F_2_ genotyping results were run in MAPMAKER 3.0 (Lander et al., 1987; Lincoln et al., 1992) with the Kosambi map function, obtaining the genetic position of each marker.

To identify markers linked to the resistance to ToLCNDV derived from the PI 604506 accession, a QTL analysis was performed using symptoms at 15 and 30 dpi and ToLCNDV relative accumulation at 30 dpi as quantitative traits, and a qualitative score of resistance (0 susceptible phenotype and 1 resistant phenotype) assigned to each plant according to symptoms and viral accumulation. We used the Kolmogorov–Smirnov test to check the normality assumption of traits distribution. Since the traits were not normally distributed, Kruskal–Wallis non-parametric test was used for QTL detection using the MapQTL version 4.1 software (Van Ooijen, 2009), considering as significant associations those with *p* < 0.05. Since 2^(–ΔCt)^ values have a skewed distribution, we used the original ΔCt data for QTL analysis. The binary qualitative trait of resistance was also analyzed by logistic regression model, with a significance level of α = 0.05.

In addition, a composite interval mapping approach (CIM, Zeng, 1994) was applied in Qgene 4.0 (Joehanes and Nelson, 2008), using the genetic map previously generated with this F_2_. The logarithm of the odds ratio (LOD) threshold was calculated using a 1,000 permutations test per trait, for *p* < 0.05. The percentage of phenotypic variance explained (*R*^2^), the additive and dominance effects, degree of dominance, and the interval position of the QTL in accordance with a 2-U LOD drop was estimated for the highest significant peak LOD. *Loci* identified with both methods (Kruskal–Wallis and CIM) were considered true QTLs of putative interest.

### Validation of the QTL of Chromosome 8 in Additional *C. moschata* Segregating Populations Derived From PI 419083, PI 604506, and PI 381814

The previous analysis allowed detecting a major QTL responsible for the resistance in chromosome 8. To confirm this QTL in additional *C. moschata*-segregating populations and to introgress the candidate region in chromosome 8 of *C. moschata* in the zucchini (*C. pepo*) background (the cucurbit crop more severely affected by ToLCNDV), a new set of 19 SNPs of the chromosome 8 candidate region was implemented in a new Agena Bioscience platform. These new SNPs were selected to be useful for both purposes. The transcriptomic sequences of PI 604506, PI 381814, and PI 419083 (obtained in the RNAseq analysis by Montero-Pau et al., 2018) were aligned to the *C. pepo* genome (Zucchini accession MU-CU-16), available at the Cucurbit Genomics Database^2^, using Bowtie2. Integrative Genomics Viewer (IGV) (Thorvaldsdóttir et al., 2013) was used to detect variations between sequences, and those polymorphic SNPs between resistant (PI 604506 and PI 381814) and susceptible (PI 419083 and MU-CU-16) genotypes were selected. This Agena platform was employed to genotype a subset of 131 plants of the previously genotyped F_2_ (PI 419083 × PI 604506), 121 of F_2_ (PI 419083 × PI 381814), 31 BC1_PI 604506_, and 73 of BC1_PI 381814_.

For further saturation of the candidate region, five additional SNPs, not integrated in the new Agena Bioscience set, were designed with the same requirements and used to genotype the F_2_ (PI 419083 × PI 604506) population by high-resolution melting (HRM) (Vossen et al., 2009). PRIMER3 software (Untergasser et al., 2012) was employed to design the oligonucleotides for the HRM analysis. The genomic positions of all these new SNPs (Agena Bioscience platform and HRM markers) and their flanking sequences are shown in Supplementary Table 2.

A new map of chromosome 8 was constructed with 24 SNP markers (3 and 16 SNPs from the first and second Agena platforms, respectively, and 5 HRM), using genotyping results of F_2_ (PI 419083 × PI 604506). MAPMAKER 3.0 (Lander et al., 1987; Lincoln et al., 1992) software and the Kosambi map function were employed to generate the new map. The genetic distances of the new map were used in a second QTL analysis, with the F_2_ (PI 419083 × PI 604506) population, following the same procedure described above. Means of symptom scores at 30 dpi of plants from F_2_ (PI 419083 × PI 381814), BC1_PI 604506_, and BC1_PI 381814_ populations classified according to the marker classes (a, b, and h for F_2_ and h and a for BC1) were analyzed by ANOVA and Bonferroni multiple range tests using STATGRAPHIC Centurion XVI.I statistic software, to evaluate differences between means, considering statistically significant differences when *p* ≤ 0.01.

### Validation of the QTL in the Interspecific Cross *C. pepo* × *C. moschata*

An interspecific cross between the ToLCNDV-susceptible *C. pepo* accession MU-CU-16 (Sáez et al., 2016) and the resistant *C. moschata* accession PI 604506 provided five F_1_ seeds that were germinated as described above. Four seedlings were moved to a greenhouse and selfed to obtain F_2_ (MU-CU-16 × PI 604506) generation. The remaining F_1_ seedling and 176 plants of F_2_ (MU-CU-16 × PI 604506) were screened by mechanical inoculation of ToLCNDV. Symptoms and viral titers were determined by the same procedure described above.

This *Cucurbita*-interspecific F_2_ population was genotyped with the new Agena Bioscience platform and the five HRM SNP markers of chromosome 8. The genotyping results were used to construct a new genetic map of chromosome 8 and to perform an additional QTL analysis as described above.

### Genomic Variation, Structural Variants, and Synteny

To obtain a more detailed view of the underlying genomic variation in the candidate region, both *C. moschata* resistant and susceptible parents (PI 604506 and PI 419083) were fully sequenced. Raw reads are deposited in the National Center for Biotechnology Information (NCBI) under BioProject PRJNA604046. Genomic DNA was obtained from fresh tissue using CTAB extraction, and a pair-end library (2 × 150 bp) was built for each accession. Libraries were sequenced as part of an Illumina HiSeq 2000 lane by Polar Genomics (Ithaca, NY, United States). Reads were cleaned using the *ngs_crumbs* software^3^ to eliminate adapters, low-quality bases (Phred quality < 25 in a 5-bp window), reads shorter than 50 bp, and duplicated sequences. Clean reads were mapped against the reference *C. moschata* genome using *bwa-mem* (Li, 2013), and variant calling was performed using Freebayes version 1.1.0 (Garrison and Marth, 2012) after filtering reads with a mapping quality cutoff MAPQ lower than 57. To study the potential effect of the genetic changes, SNPs were annotated based on its predicted effect on the gene using SNPEff v4.3 (Cingolani et al., 2012). Differences in sequencing genome coverage between both accessions were studied to explore possible genomic deletions. Read coverage along the candidate region was calculated using samtools v.1.9 (Li et al., 2009), and we checked if coverage deviated from the 99% confidence interval of the observed coverage for each accession assuming a log-normal distribution. Confidence interval for the log-normal distribution was calculated using the function *elnorm* of R package “EnvStats” (Millard, 2013). In addition to that, the structural variant caller Manta v.1.6 (Chen et al., 2016) was used to check for differential large insertion/deletions. Identification of putative paralogs of the genes in the candidate region was done with OrthoMCL (Li et al., 2003).

Identification of syntenic regions between *C. moschata* and *C. pepo* and *C. melo* was done by nucleotide basic local alignment search tool (BLAST) of each gene within the candidate region of *C. moschata* against the other two genomes. BLAST hits were filtered using an *E* value cutoff of 10^–20^ and a minimum overlap between sequences of 70%. For *C. pepo*, to inspect for possible insertion/deletions, a dot plot comparing chromosome 17 region of *C. pepo* and chromosome 8 of *C. moschata* was built based on the alignment of both sequences using LAST (Kielbasa et al., 2011). For *C. melo*, the module of Tripal “SyntenyViewer,” available in cucurbitgenomics.com, was used to visualize the synteny.

New *C. moschata* and *C. pepo* genome assemblies have become recently available^4^ ^,5^ (online availability since November 2019), but after finishing the analysis that we showed here. Our results were checked through alignments with the new assemblies to avoid misinformation.

In addition to the analysis of the genomic sequences, SNPs discovered using the available RNAseq data (Montero-Pau et al., 2018) from the three *C. moschata* accessions used as parentals in the previous crosses and six additional *C. moschata* from different origins that exhibited susceptibility to ToLCNDV in previous works (López et al., 2015; Sáez et al., 2016) were also annotated using SNPEff.

## Results

### Response to ToLCNDV of F_1_ Progenies

**FIGURE 1:**
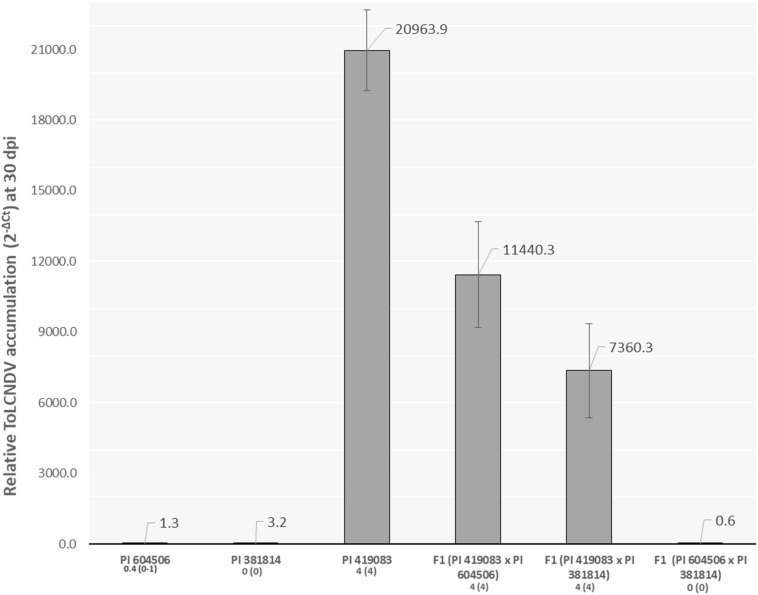
Relative *tomato leaf curl New Delhi virus* (ToLCNDV) accumulation (calculated as 2^– ΔCt^) at 30 days after mechanical inoculation (dpi) with ToLCNDV in the resistant *Cucurbita moschata* accessions PI 604506 and PI 381814, the susceptible control PI 419083, and their respective hybrids. Mean and range of symptoms scores at 30 dpi are indicated in the *x*-axis legend.

The inoculation assay showed that the two *C. moschata* accessions resistant to ToLCNDV, PI 604506 and PI 381814, remained totally symptomless or with only slight symptoms (score from 0 to 1) at 30 dpi, contrasting with the severe mosaic developed in the susceptible control (score 4), PI 419083 (Figure 1). F_1_ plants of the two susceptible × resistant crosses were highly susceptible, displaying a similar symptomatology as PI 419083 at 30 dpi. Conversely, F_1_ progeny derived from the cross between the two *C. moschata*-resistant accessions remained symptomless throughout the essay (Figure 1).

Strong correlation between symptom severity and viral titers was observed (*r*^2^ = 0.73, *p* = 0.030) after measuring relative ToLCNDV accumulation by qPCR. In accordance with their resistant behavior, PI 604506, PI 381814, and the F_1_ (PI 604506 × PI 381814) had viral titers, on average, 7.8 × 10^3^ times lower than those of the susceptible control PI 419083 and the two F_1_ derived from it (Figure 1).

The fact that F_1_ progenies derived from the two susceptible × resistant crosses were susceptible, while the F_1_ derived from the resistant × resistant cross was resistant, suggests a recessive genetic control of the resistance in both accessions, controlled by common genes. A further analysis of the genetic control of the resistance was performed in F_2_ and BC1 populations.

### Response to ToLCNDV of Segregating Populations Derived From the Cross Between Resistant and Susceptible *C. moschata* Accessions

**FIGURE 2:**
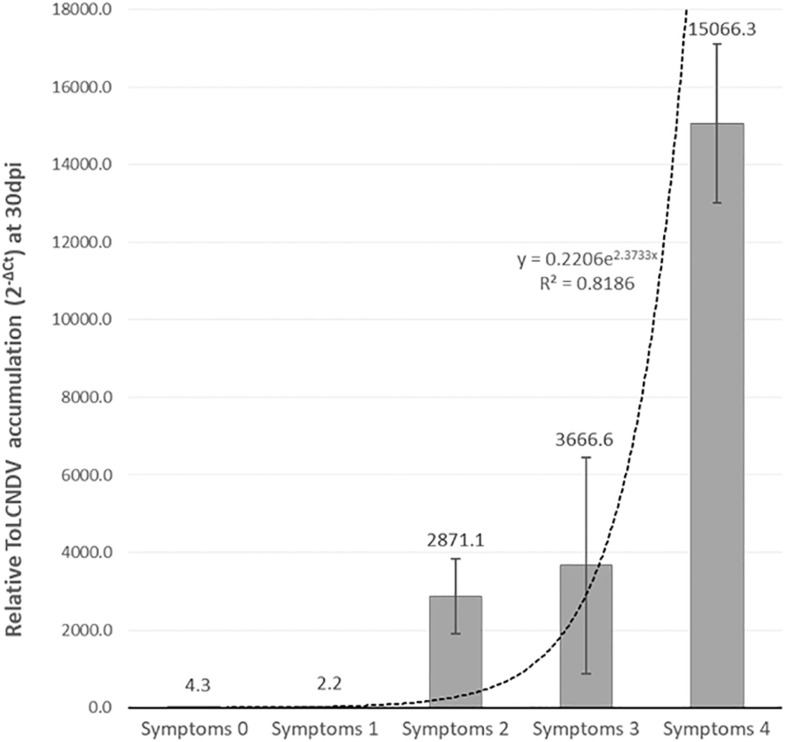
Mean of relative *tomato leaf curl New Delhi virus* (ToLCNDV) accumulation (calculated as 2^– ΔCt^) in plants within each symptomatic class in F_2_ (PI 419083 × PI 604506) at 30 days after mechanical inoculation. Dot line represents the exponential relationship between both variables, which was statistically significant for a confident level of α = 0.05.

F_2_ and BC1_PI 604506_ populations, derived from the F_1_ PI 419083 × PI 604506, segregated for symptoms severity. Table 1 shows resistant:susceptible plants segregation, according to symptomatology at 30 dpi. At the end of the assay, 38 plants of F_2_ remained symptomless (score 0), and 96 showed severe symptomatology (scores 2–4). The *X*^2^ test indicated that this segregation fitted to a 1:3 (resistant:susceptible) ratio expected for a single recessive gene for resistance (*p* = 0.43) (Table 1). To further characterize the response to ToLCNDV, virus accumulation was estimated in the segregating population F_2_ (PI 419083 × PI 604506) by qPCR (Figure 2). On average, viral titer strongly correlated to symptoms severity following an exponential model (*r*^2^ = 0.82, *p* = 0.035). All plants developing mosaic, deformation, or short internodes had high viral titers, whereas in the symptomless plants, ToLCNDV accumulation was detected at very low concentrations. On average, the viral accumulation (2^–ΔCt^) in susceptible plants was 2.2 × 10^3^ times higher than in resistant plants. Since viral titer is in concordance with symptoms development, symptom scores were used to phenotype the response to ToLCNDV in plants of the remaining F_2_ and BC1 populations. In BC1_PI 604506_, 33 plants were resistant (score 0) and 26 were susceptible (scores 2–4). This segregation also fitted to a 1:1 ratio expected for a single recessive gene (*p* = 0.44) (Table 1). In accordance with the occurrence of a single recessive gene controlling the resistance, all plants of the BC1_PI 419083_ generation had severe symptoms at the end of the assay.

**TABLE 1:** Segregation of resistant/susceptible plants in F_2_ and BC progenies at 30 days after mechanical inoculation with *tomato leaf curl New Delhi virus* (ToLCNDV).

			**Resistant:**		
			**susceptible**		
**Female parent**	**Male parent**	**Progeny**	**segregation**	**Ratio**	***X*^2^test^a^**
PI 419083 (S)^b^	PI 604506 (R)^b^	F_2_	38:96	1:3	0.6368 (*p* = 0.43)
PI 419083 (S)	PI 604506 (R)	BC_PI 419083_	0:90	0:1	–
PI 419083 (S)	PI 604506 (R)	BC_PI 604506_	33:26	1:1	0.6102 (*p* = 0.44)
PI 419083 (S)	PI 381814 (R)	F_2_	40:81	1:3	3.7713 (*p* = 0.047)
PI 419083 (S)	PI 381814 (R)	BC_PI 381814_	43:30	1:1	1.9726 (*p* = 0.16)
PI 604506 (R)	PI 381814 (R)	F_2_	160:0	1:0	–

*^*a*^Probability of the χ^2^ value calculated for a recessive monogenic expected ratio. ^*b*^(S), susceptible genotype; (R), resistant genotype.*

Symptom segregation ratios observed in the F_2_ (PI 419083 × PI 381814) and BC1_PI 381814_ populations also fitted to one recessive gene for resistance null hypothesis in *X*^2^ test (Table 1). Forty and 43 plants of F_2_ and BC1_PI 381814_, respectively, remained symptomless (score 0), and 81 and 30 plants showed severe symptoms (scores 2–4), with *p* = 0.047 and *p* = 0.16, in both respective populations (Table 1).

In accordance with the F_1_ results, the 160 plants of the F_2_ derived from the resistant × resistant cross PI 604506 × PI 381814 remained totally symptomless along all the assay.

### QTL Analysis in F_2_ (PI 419083 × PI 604506) Population

The F_2_ (PI 419083 × PI 604506) population was genotyped with the 137 SNPs markers evenly distributed throughout *C. moschata* genome and used to construct a linkage map that included 20 linkage groups and spanned a total of 2,681.5 cM of genetic distance, with an average of 22.92 cM between markers (Supplementary Table 1). The linkage map was used to identify QTLs involved in ToLCNDV resistance in *C. moschata*, based on genotyping and phenotyping results (symptoms scores at 15 and 30 dpi, virus titer at 30 dpi, and the qualitative resistance score) of F_2_ (PI 419083 × PI 604506) population. QTL analysis, performed using non-parametric Kruskal–Wallis test (KW) followed by CIM, resulted in the detection of a major QTL in chromosome 8 (Table 2), validated by logistic regression of the qualitative trait of resistance (data not shown). Four QTLs, all located in almost the same genetic position, showed significant association with all the traits evaluated, explaining a proportion between 29.0 and 45.0% of the observed phenotypic variance. All QTLs (ToLCNDVCm_Sy15-8, ToLCNDVCm_Sy30-8, ToLCNDVCm_VT30-8, and ToLCNDVCm_Re-8) were located close to D133 (physical position, 1,366,729 bp), with LOD peaks between 10.06 and 17.31.

**TABLE 2:** Quantitative trait loci (QTLs) identified in the F_2_ (PI 419083 × PI 604506) segregating population genotyped with the set of 137 single nucleotide polymorphisms (SNPs) evenly distributed through the *C. moschata* genome, using the non-parametric Kruskal–Wallis test and composite interval mapping method.

			Kruskal–Wallis							
			
			cM (peak		Nearest					
Trait	Chr^a^	QTL name	position)		marker^b^	*K*^c^	Mean a^d^	Mean h^e^		Mean b^f^
Symptoms at 15 dpi	8	ToLCNDVCm_Sy15-8	0		D133	35.51	2.78	2.20		0.46
Symptoms at 30 dpi	8	ToLCNDVCm_Sy30-8	0		D133	55.88	4.00	3.44		1.10
Viral titer at 30 dpi (ΔCt)	8	ToLCNDVCm_VT30-8	0		D133	33.11	–12.76	–10.67		4.24
Resistance (qualitative trait)	8	ToLCNDVCm_Re-8	0		D133	59.14	0.00	0.13		0.73

			**CIM (Qgene)**							
			
			**cM (peak**	**Interval**	**Nearest**					
**Trait**	**Chr^a^**	**QTL name**	**position)**	**(cM)^g^**	**marker^b^**	**LOD^h^**	**Add^i^**	**Dom^j^**	**(*d*/*a*)*^k^***	***R*^2(l)^**

Symptoms at 15 dpi	8	ToLCNDVCm_Sy15-8	4.00	0–21.27	D133	10.06	1.52	0.84	0.55	0.29
Symptoms at 30 dpi	8	ToLCNDVCm_Sy30-8	0.00	0–5.43	D133	16.52	1.45	0.90	0.62	0.44
Viral titer at 30 dpi (ΔCt)	8	ToLCNDVCm_VT30-8	0.00	0–6.82	D133	13.97	–4.26	–2.20	0.52	0.39
Resistance (qualitative trait)	8	ToLCNDVCm_Re-8	0.00	0–5.17	D133	17.31	–0.37	–0.23	0.64	0.45

^*a*^Chromosome. ^*b*^The closest marker to LOD peak. ^*c*^K*: the Kruskal–Wallis test statistic, with a significant level of 0.0001. ^*d*^Mean of the PI 419083 genetic class in each marker. ^*e*^Mean of the PI 419083/PI 604506 genetic class in each marker. ^*f*^Mean of the PI 604506 genetic class in each marker. ^*g*^Interval position of the putative QTL, identified in the F_2_ (PI 419083 × PI 604506) in cM on the genetic map according with a LOD drop of 2. ^*h*^*LOD* higher logarithm of the odds score. ^*i*^*Add* additive effect of the PI 419083 allele. ^*j*^*Dom* dominant effect of the PI 419083 allele. ^*k*^*d/a* degree of dominance. ^*l*^*R*^2^ percentage of phenotypic variance explained by the QTL.

### Narrowing the Candidate Region in Chromosome 8

**FIGURE 3:**
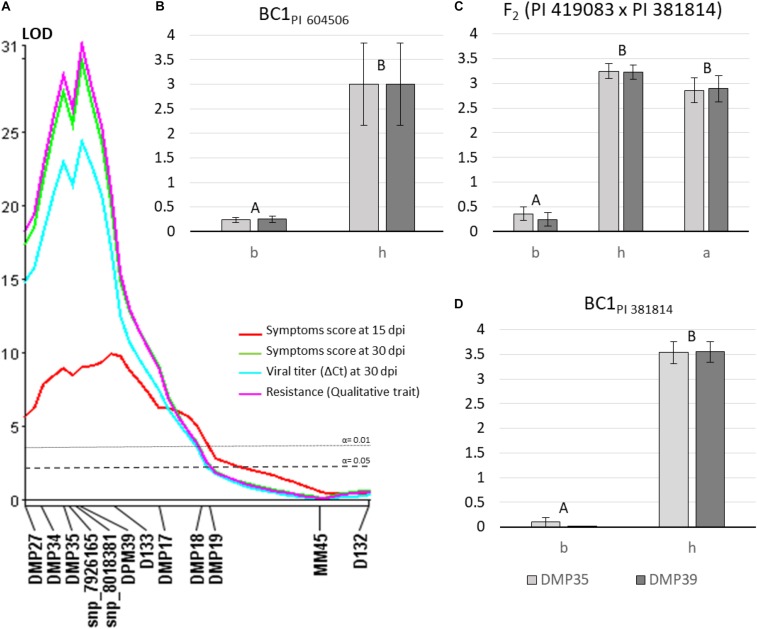
**(A)** Molecular markers linked to quantitative trait loci (QTLs) for the four traits [symptoms at 15 and 30 days after mechanical inoculation (dpi), virus titer at 30 dpi and the qualitative resistance trait] associated with ToLCNDV resistance. QTL location was obtained by composite interval mapping (CIM) method using F_2_ (PI 419083 × PI 604506). **(B–D)** Mean of symptom score at 30 dpi in BC_PI 604506_, F_2_ (PI 419083 × PI 381814) and BC_PI 381814_ populations, respectively, according to each genotypic class DMP35 (light gray bars) and DMP39 (dark gray bars) markers (chromosome 8). PI 604506 or PI 381814 homozygous genotype is represented as “b,” heterozygous as “h” and PI 419083 homozygous plants as “a.” Bars with same capitals letters are not significantly different at *p* ≤ 0.01.

To validate the major QTL identified in the previous analysis and to increase marker density in the candidate region, F_2_ (PI 419083 × PI 604506) population was genotyped with the new Agena Bioscience-HRM SNPs set of chromosome 8. Twenty-one out of the 24 new markers (Supplementary Table 2) were polymorphic in this population, despite all of them were selected *in silico* as SNP variants between both parents using the IGV software. Genotyping results were employed to generate a new linkage map in this region (Supplementary Table 2), covering 72.5 cM, with an average distance between consecutive markers of 3.15 cM, and two clusters of linked markers at 0 and 11.7 cM genetic positions. The QTL analysis was performed using the new map and the new genotyping results (using one selected marker of each of the two clusters of completely linked SNPs) (Table 3). ToLCNDVCm_Sy15-8 QTL, associated to the variation of symptoms at 15 dpi, was identified again with both non-parametric Kruskal–Wallis and CIM analysis, near D133 (located at 18.8 cM in this new map) and with similar explained variance, LOD peak, additive, and dominant effects. However, ToLCNDVCm_Sy30-8, ToLCNDVCm_VT30-8, and ToLCNDVCm_Re-8 QTLs, corresponding to traits measured at the end of the assay (30 dpi), when differences are clearer between resistant and susceptible plants, were closely linked to a new marker with both analysis methods. The closest markers (those linked at 11.7 cM DPM37, DMP39, DMP11, DMP10, DMP42, DMP43, DMP44, DMP41, and snp_8202510 markers) are included in the interval position of the same QTLs identified with Kruskal–Wallis and CIM (between 8 and 14 cM) (Figure 3A) and validated with logistic regression of the qualitative trait of resistance, according to their physical and genetic position. The interval of the four QTLs was flanked by DPM34 and D133 markers, with physical positions 561,788 and 1,366,729 bp, respectively. After this further QTL analysis of chromosome 8, the proportion of explained variance was increased, with percentages between 29.5 and 66.0% of *R*^2^.

**TABLE 3:** Quantitative trait loci (QTLs) identified in the F_2_ (PI419083 × PI604506) segregating population genotyped with markers of chromosome 8 of *C. moschata*, using the non-parametric Kruskal–Wallis test and composite interval mapping (CIM).

			Kruskal-Wallis					
			
			cM (peak		Nearest					
Trait	Chr^a^	QTL name	position)		marker^b^	K^c^	Mean a^d^	Mean h^e^		Mean b^f^
Symptoms 15 dpi	8	ToLCNDVCm_Sy15-8	18.8		D133	35.58	2.84	2.22		0.46
Symptoms 30 dpi	8	ToLCNDVCm_Sy30-8	11.7		DMP39^m^	82.96	4.00	3.60		0.46
Viral titer at 30 dpi (ΔCt)	8	ToLCNDVCm_VT30-8	11.7		DMP39^m^	50.38	−12.63	−11.14		−2.42
Resistance (qualitative trait)	8	ToLCNDVCm_Re-8	11.7		DMP39^m^	86.71	0.00	0.10		0.89
m: markers with same genetic position and significance in the analysis: DPM37, DMP39, DMP11, DMP10, DMP42, DMP43, DMP44, DMP41								

			**CIM (Qgene)**							
			
			**cM (peak**	**Interval**	**Nearest**					
**Trait**	**Chr^a^**	**QTL name**	**position)**	**(cM)^g^**	**marker^b^**	**LOD^h^**	**Add^i^**	**Dom^j^**	**[d/a]^k^**	***R*^2 l^**

Symptoms 15 dpi	8	ToLCNDVCm_Sy15-8	18.00	4.53–24.00	D133	9.96	1.25	0.60	0.48	0.30

Symptoms 30 dpi	8	ToLCNDVCm_Sy30-8	12.00	8.01–13.46	DMP39	29.76	1.79	1.38	0.77	0.65
Viral titer at 30 dpi (ΔCt)	8	ToLCNDVCm_VT30-8	12.00	10.85–14.15	DMP39	24.37	−5.18	−3.64	0.70	0.59
Resistance (qualitative trait)	8	ToLCNDVCm_Re-8	12.00	8.13–13.83	DMP39	30.92	−0.45	−0.35	0.76	0.66

Genetic positions are according with the new *C. moschata* × *C. moschata* linkage map of chromosone 8. ^*a*^Chromosome. ^*b*^The closest marker to LOD peak. ^*c*^K*: the Kruskal–Wallis test statistic, with a significant level of 0.0001. ^*d*^Mean of the PI 419083 genetic class in each marker. ^*e*^Mean of the PI 419083/PI 604506 genetic class in each marker. ^*f*^Mean of the PI 604506 genetic class in each marker. ^*g*^Interval position of the putative QTL, identified in the *F*_2_ (PI 419083 × PI 604506) in cM on the genetic map according with a LOD drop of 2. ^*h*^*LOD*, higher logarithm of the odds score. ^*i*^*Add*, additive effect of the PI 419083 allele. ^*j*^*Dom*, dominant effect of the PI 419083 allele. ^*k*^*d/a*, degree of dominance. ^*l*^*R*^2^ percentage of phenotypic variance explained by the QTL.

### Validation of the Major QTL in Chromosome 8 in BC1_PI 604506_, F_2_ (PI 419083 × PI 381814) and BC1_PI 381814_ Segregating Populations

The Agena Bioscience SNPs panel of chromosome 8 was used to genotype the BC1_PI 604506_ derived from the PI 419083 × PI 604506 cross, and the F_2_ and BC1_PI 381814_ populations derived from the PI 419083 × PI 381814 cross. Mean of symptoms scores at 30 dpi were calculated for each genotypic class of selected SNPs located within the defined QTL interval (DMP35 and DMP39) and compared in Figures 3B–D. The lowest level of symptoms was observed when plants in the three populations had the PI 604506 or PI 381814 homozygous genotype (b), in DPM35 or DPM39 indistinctly. Plants heterozygous (h) or PI 419083 homozygous (a) in both markers displayed significantly more severe symptomatology.

### QTL Analysis and Validation of the Candidate Region in *C. pepo*

**FIGURE 4:**
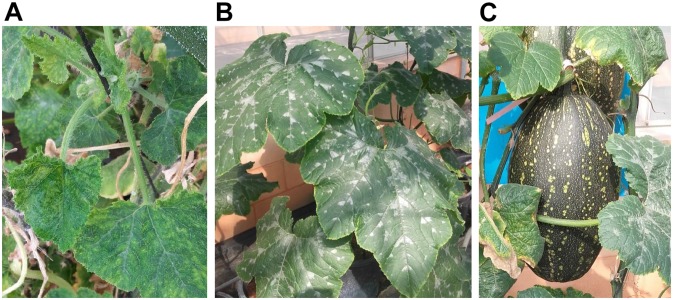
Plants of the interspecific F_1_ resulting from the cross *C. pepo* MU-CU-16 × *C. moschata* PI 604506. **(A)** Typical symptoms of *tomato leaf curl New Delhi virus* including curling, severe mosaic, and short internodes. **(B)** Healthy plant used to obtain F_2_ progeny by selfing in a greenhouse. **(C)** Detail of F_1_ (MU-CU-16 × PI 604506) fruit obtained by selfing.

Consistently with the results obtained in F_1_ from susceptible × resistant *C. moschata* crosses, severe symptoms were developed by F_1_ *C. pepo* MU-CU-16 × *C. moschata* PI 604506 plants at 15 and 30 dpi (Figure 4). This result supports that resistance in PI 604506 has a recessive genetic control. F_2_ (MU-CU-16 × PI 604506) plants segregated for symptomatology and viral accumulation. Symptoms, including upward and downward curling and severe mosaic of young leaves, short internodes, and bad distorted development, were observed in 124 and 151 F_2_ (MU-CU-16 × PI 604506) plants at 15 and 30 dpi, respectively. The number of resistant plants decreased from 52 to 25 between 15 and 30 dpi. Nine plants had bad development or died in the course of the infection. On average, virus titers determined by qPCR at 30 dpi were in concordance with symptoms development, with mean of relative viral accumulation expressed as 2^(–ΔCt)^ of 1.04 ± 0.31 and 49,571.67 ± 9,670.31 in resistant and susceptible plants, respectively. The observed segregation proportion was adjusted to the expected ratio resistant/susceptible plants, in case of one recessive gene responsible on the genetic control of resistance to ToLCNDV at 15 dpi (*X*^2^ = 2.1894, *p* = 0.14), but not at 30 dpi (*X*^2^ = 10.312, *p* = 0.0014).

The genetic map of chromosome 8 generated with the genotyping results of the Agena Bioscience-HRM SNPs in the F_2_ (MU-CU-16 × PI 604506) gave a total genetic length of 21.4 cM, with an average genetic distance between successive markers of 0.98 cM (Supplementary Table 2).

The QTL analysis performed in this population show that the QTLs identified in the *C. moschata* populations were stable in the cross with the *C. pepo* accession MU-CU-16. ToLCNDVCm_Sy15-8, ToLCNDVCm_Sy30-8, ToLCNDVCm_ VT30-8, and ToLCNDVCm_Re-8 were located in the same region that in *C. moschata* (Table 4), physically mapped in chromosome 17. The highest *R*^2^ value (65%) was explained by the ToLCNDVCm_Sy15-8, associated to DMP39 as the nearest marker to the peak LOD. *R*^2^ values were lower in QTLs related to advanced stages of the ToLCNDV infection, mainly in the viral titer at 30 dpi (ΔCt) trait. In these cases, the nearest markers to the LOD peaks were DMP39 and snp_7926165 in ToLCNDVCm_Sy30-8 (Kruskal–Wallis and CIM tests, respectively), snp_7926165 in ToLCNDVCm_VT30-8 and DMP35, and snp_7926165 in ToLCNDVCm_Re-8 (Kruskal–Wallis and CIM tests, respectively). Logistic regression validate the occurrence of ToLCNDVCm_Re-8 QTL. According to the 2-LOD drop confidence intervals, the position interval where the four QTLs are comapping in chromosome 17 of *C. pepo* genome (v.4.1) is delimited between DMP34 (7,658,175 bp) and DMP41 (8,165,929).

**TABLE 4:** Quantitative trait loci (QTLs) identified in the F_2_ (MU-CU-16 × PI 604506) segregating population genotyped with markers evenly distributed in chromosome 8 of *C. moschata*, using the genetic map obtained with this population, using the non-parametric Kruskal–Wallis test and composite interval mapping (CIM).

		Kruskal–Wallis							
			
			cM (peak		Nearest					
Trait	Chr^a^	QTL name	position)		marker^b^	*K*^c^	Mean a^d^	Mean h^e^		Mean b^f^
Symptoms 15 dpi	17	ToLCNDVCm_Sy15-8	10.5		DMP39	109.65	3.78	3.49		0.25
Symptoms 30 dpi	17	ToLCNDVCm_Sy30-8	10.5	DMP39	75.99	3.87	3.84	1.73
Viral titer at 30 dpi (ΔCt)	17	ToLCNDVCm_VT30-8	8.3		snp_7926165	28.89	–14.51	–13.83		–6.02
Resistance (qualitative)	17	ToLCNDVCm_Re-8	7.2		DMP35	63.18	0.02	0.02		0.53

			**CIM (Qgene)**							
			
			**cM (peak**	**Interval**	**Nearest**					
**Trait**	**Chr^a^**	**QTL name**	**position)**	**(cM)^g^**	**marker^b^**	**LOD^h^**	**Add^i^**	**Dom^j^**	**(*d*/*a*]^k^**	***R*^2l^**

Symptoms 15 dpi	17	ToLCNDVCm_Sy15-8	10	8.22–10.70	DMP39	40.57	1.76	1.49	0.84	0.65
Symptoms 30 dpi	17	ToLCNDVCm_Sy30-8	8	6.98–11.32	snp_7926165	20.70	1.08	1.05	0.97	0.42
Viral titer at 30 dpi (ΔCt)	17	ToLCNDVCm_VT30-8	8	6.57–11.62	snp_7926165	15.32	–4.25	–3.56	0.84	0.33
Resistance (qualitative)	17	ToLCNDVCm_Re-8	8	6.92–11.33	snp_7926165	17.28	–0.25	–0.25	1.00	0.37

^*a*^Chromosome. ^*b*^The closest marker to LOD peak. ^*c*^K*, the Kruskal–Wallis test statistic, with a significant level of 0.0001. ^*d*^Mean of the MU-CU-16 genetic class in each marker. ^*e*^Mean of the MU-CU-16/PI 604506 genetic class in each marker. ^*f*^Mean of the PI 604506 genetic class in each marker. ^*g*^Interval position of the putative QTL, identified in the *F*_2_ (MU-CU-16 × PI 604506) in cM on the genetic map according with a LOD drop of 2. ^*h*^*LOD* higher logarithm of the odds score. ^*i*^*Add* additive effect of the MU-CU-16 allele. ^*j*^*Dom* dominant effect of the MU-CU-16 allele. ^*k*^*d/a* degree of dominance. ^*l*^*R*^2^ percentage of phenotypic variance explained by the QTL.

After both QTL analysis of chromosome 8, a consensus candidate region considered as responsible for ToLCNDV resistance in *C. moschata*, was established between DMP34 (561,788) and snp_8202510 (1,116,660).

### Genomic Variation, Structural Variants, and Synteny

The alignment between the reference assemblies of *C. moschata* and *C. pepo* used for mapping purposes in the current paper^6^ and the new assemblies available in November 2019^7^ showed no significant effect on the QTL region studied here (Supplementary Figure 1). Consequently, we keep working with the previous reference versions of both genomes.

**FIGURE 5:**
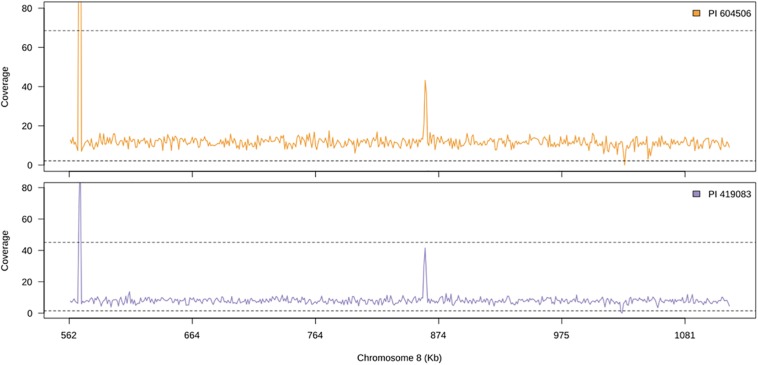
Genomic coverage along candidate region of chromosome 8 of the two accession used as parents for quantitative trait locus (QTL) mapping. Solid line shows the average coverage for 1-kb windows. Dashed line shows the upper and lower 99% confidence interval for the observed coverage for the whole genome.

A total of 53.2 and 31.5 million genomic clean reads were obtained from PI 604506 and PI 419083, respectively, and approximately more than 97% of them mapped against the *C. moschata* v.1 reference genome. No large structural variants were found between both accessions, and the read genome coverage was similar among them (Figure 5), which indicates that there are no deletions causing the observed phenotype. Some genomic positions show significant deviations for the expected coverage in both accessions (Figure 5), which could indicate some assembly errors on the reference genome.

After filtering for mapping quality, 28.2 and 18.6 million reads were kept. A total of 1,220,940 SNPs were found to be variable between both parental accessions, and 2,748 were located in the candidate region in chromosome 8. Out of them, nine SNPs had a predicted high impact (either a frameshift or missense variant, a stop codon gain/loss, or a splice site variant) and located within six genes (Supplementary Table 3). Two of these markers are located in the same genes where SNPs used in mapping (snp_7926165 and DMP44) were detected to be linked to ToLCNDV resistance [CmoCh08G001470 encoding a BZIP transcription factor bZIP80 (835,327 to 841,749 bp) and CmoCh08G001770 encoding an unknown protein (1,047,526–1,051,835 bp)]. The remaining seven SNPs with predicted high impact were located in three additional genes of this interval [CmoCh08G001130 encoding a Ribosome inactivating protein (583,200–588,238 bp), CmoCh08G001780 encoding a putative transmembrane protein (1,051,479–1,053,847 bp) and CmoCh08G001880 coding a IQ-DOMAIN 14-like protein (1,097,864–1,102,974)]. In addition, some other SNPs with low, moderate, or unknown modifying effect are placed in genes related to plant virus resistance (Supplementary Table 3).

In addition to the genomic SNPs, the transcriptomic sequences of the three parentals and the six additional susceptible *C. moschata* accessions provided 731 SNPs in the candidate region, 94 of them were fixed for different alleles in the PI 604506-resistant accession and in the seven susceptible accessions (Supplementary Table 3). PI 381814 transcriptomic sequence had a low coverage in the candidate region, and it was not possible to identify common polymorphisms between the two resistant accessions, PI 604506 and PI 381814. Three SNPs were detected with high predicted effect, all of them were common to those found in the genomic sequences analyzed and were located in three genes (Supplementary Table 3) (CmoCh08G001130 encoding a ribosome-inactivating protein, CmoCh08G001470 encoding a BZIP transcription factor bZIP80 and, CmoCh08G001770 encoding an unknown protein).

**FIGURE 6:**
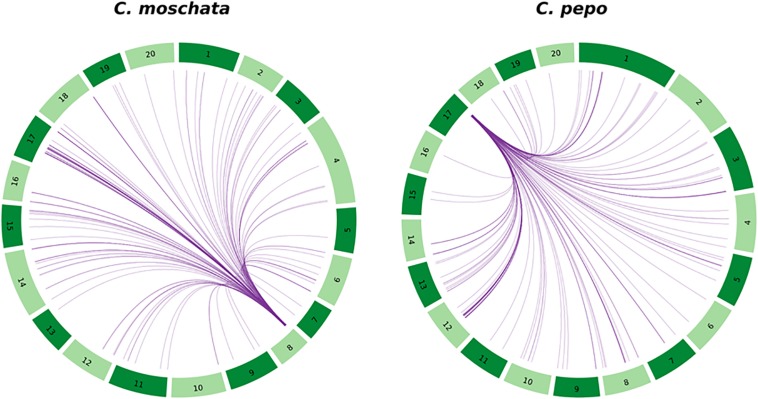
Circos plot showing the location of the duplicated genes located in *C. moschata*’s candidate region of chromosome 8 and in *C. pepo*’s syntenic region to *C. moschata* located in chromosome 17.

The structure of the candidate region was studied in more detail. A whole genome duplication likely occurred in the species that originated the *Cucurbita* genus (Montero-Pau et al., 2018). In fact, the search for putative paralogs of the genes in the chromosome 8 region indicated that 68 out of the 86 genes in the chromosome 8 candidate region could be assigned to an orthogroup, and 58 of them presented at least one paralog gene. These paralog genes are widespread along the genome (Figure 6), although it seems that there is a conserved duplicated region of chromosome 8 on chromosome 17. Interestingly, some genes of the candidate region have been identified as single copy in chromosome 8 (Supplementary Table 4), without paralog genes in other chromosomes, which is consistent with a major QTL responsible of ToLCNDV resistance.

**FIGURE 7:**
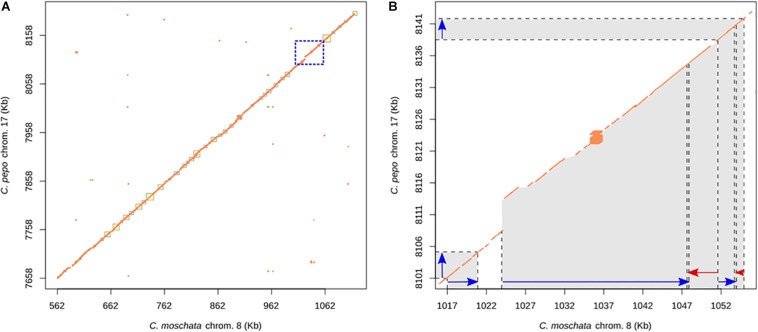
**(A)** Dot plot showing the alignment between the chromosome 8 of *C. moschata* assembly v.1 and chromosome 17 of *C. pepo* v.4.1. **(B)** Expanded syntenic region where large INDELs have been detected. Blue and red arrows points genes sense.

We also studied the synteny of this region with the susceptible *C. pepo*, which is phylogenetically closely related to *C. moschata.* BLAST alignment showed synteny between chromosome 8 region and chromosome 17 from 7,658,023 to 8,205,474 bp of *C. pepo* (see Figure 7 and Supplementary Table 4). Gene order and orientation is preserved for most genes, but there is one region showing INDELs. Interestingly, the region with a major insertion in *C. pepo*, from 8,108,962 to 8,113,419 bp, is the region in which the MAD-box transcription factor CmoCh08G001760 maps. This region correspond to position 1,024,011 bp of *C. moschata*, located between the 5′ untranslated region (UTR) and the first exonic region of this gene. Specific analysis of this *C. pepo* insertion sequence allowed to detect a long terminal repeats (LTR) retrotransposon of Ty1-copia Retrofit/Ale kind, of 3,692 bp length located from 8,109,186 to 8,113,548 bp. This transposable sequence was previously annotated using the annotation procedure for repetitive sequences described in Montero-Pau et al. (2018). Supplementary Table 6 shows the annotation results and the fasta sequence of the region. Although this insertion is absent in both resistant (PI 604506) and susceptible (PI 419083) *C. moschata* accessions (Figure 5), many polymorphic SNPs between them are located in this gene, including 5′ UTR and 3′ UTR variants (1,023,872 and 1,047,775 bp), and a missense variant with moderate effect (1,043,369).

**FIGURE 8:**
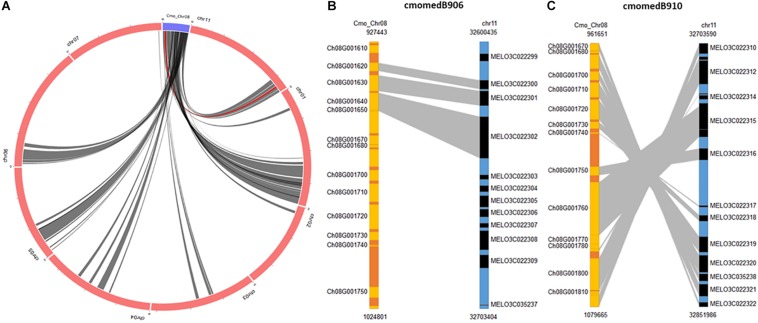
Synteny between the interval regions of quantitative trait loci (QTLs) detected in chromosome 8 of *C. moschata* and the major QTL in chromosome 11 of *C. melo.* **(A)** Circular representation showing synteny with genome of melon DHL92 (v3.6.1). **(B,C)** Synteny blocks where QTLs linked to *tomato leaf curl New Delhi virus* (ToLCNDV) resistance are located (coded as cmomedB906 and cmomedB910 in the database).

BLAST search of the *C. moschata* QTL region against *C. melo* found several syntenic regions. In the case of *C. melo*, highly significant alignments were obtained against chromosome 11 where a major QTL associated with resistance to ToLCNDV is located (Sáez et al., 2017). Results show inversions in SNPs positions between both species, with at least two points of inversion events and loss of information regions (Supplementary Table 2). This syntenic relationship was confirmed with the information displayed by the SyntenyViewer of cucurbitgenomics.org tool. Using chromosome 8 of *C. moschata* as query genome and location, circular representation showed regions of synteny with eight chromosomes of melon DHL92 (v3.6.1), including the candidate region of chromosome 11 (Figure 8A). Figures 8B,C show the syntenic blocks where ToLCNDV resistance-linked QTLs are located (coded as cmomedB906 and cmomedB910 in the database), the genomic position covered, and the graphic synteny relationship in both blocks. Furthermore, statistical significance of synteny between homologous genes in the candidate region of *C. moschata* and *C. melo* is presented in Supplementary Table 5. Seventeen genes are shared by both candidate regions, including the MAD-box transcription factor CmoCh08G001760 and the transmembrane protein CmoCh08G001780 where INDELs or high-effect SNPs have been identified.

## Discussion

In this work, we evaluated the resistance to ToLCNDV previously described in the two *C. moschata* accessions PI 604506 and PI 381814 using mechanical inoculation (Sáez et al., 2016). Our results confirmed that both genotypes remain symptomless after inoculation assays. The Large Cheese improved cultivar PI 604506 originated in the United States (Burpee Company). Even though the primary center of *C. moschata* diversity is located in Northern South America and Central America, it spread soon to Mexico and later to the Caribbean area and the United States, where it diversified (Decker-Walters and Walters, 2000). The landrace PI 381814 was collected in India, a secondary center of *C. moschata* variation, where resistance to ToLCNDV was found in melon accessions (López et al., 2015). This fact can be related with the coevolution of host and pathogen in this area, in which ToLCNDV was detected for the first time infecting cucurbits many years ago. Indian cucurbits germplasm has been previously used as source of resistances to viral and fungal pathogens (Dhillon et al., 2012; McCreight et al., 2017). Mendelian analysis of symptom segregation in F_2_ and BC_1_s populations derived from PI 604506 and PI 381814, as well as QTL results, suggested the presence of a major recessive gene in chromosome 8 of *C. moschata* controlling symptoms development and virus titer. Allelism test results, which show resistance in all plants of F_2_ (PI 604506 × PI 381814), suggests that alleles of the same *locus* control ToLCDV resistance in both accessions.

The occurrence of a major gene controlling ToLCNDV resistance derived from *C. moschata* sources is consistent with the existence of a major QTL reported to control the resistance to ToLCNDV in melon, derived from the wild Indian accession of *Cucumis melo* subsp. *agrestis* WM-7 (Sáez et al., 2017). Resistance to whitefly transmission of ToLCNDV in sponge gourd (*L. cylindrica*), a cucurbit crop widely cultivated in India (Islam et al., 2010), has also been described to be regulated by a main dominant gene, for which two linked sequence-related amplified polymorphism (SRAP) markers were reported (Islam et al., 2011).

Even though the major QTL linked to the resistance in *C. moschata* was stable in the *C. pepo* × *C. moschata* interspecific progeny, the mendelian segregation of symptoms only fitted to one recessive gene at 15 dpi. The effect of additional minor genes contributing to ToLCNDV resistance that are segregating in this interspecific population could account for these differences. In fact, in melon, besides the major QTL of chromosome 11, two additional minor regions in chromosomes 12 and 2 modifying the resistant response were identified (Sáez et al., 2017). In a recent publication (Romay et al., 2019), one recessive (*bgm-1*) and two dominant (*Bgm-2* and *Tolcndv*) genes were also found controlling resistance to ToLCNDV in the same Indian accession WM-7. A similar oligogenic control, three dominant genes, has been reported in *S. habrochaites* S. Knapp & D. M. Spooner, a wild species related to tomato, after ToLCNDV agroinoculation (Rai et al., 2013).

The role of the genetic background in resistance to plant viruses is considered determinant in breeding programs when transferring QTLs from one species to another. Gallois et al. (2018) have studied and reviewed the effect of epistatic relationship with QTL analysis on virus resistance, suggesting that a major-effect QTL (proportion of phenotypic variance explained by the QTL *R*^2^ > 0.60) could be more susceptible to genetic background influence than minor-effect QTLs. This statement supports the incomplete penetrance obtained when we tried to transfer the QTL conferring resistance to ToLCNDV from chromosome 8 of *C. moschata* into *C. pepo* background. In this work, *R*^2^ percentages of QTLs detected in the F_2_ (PI 419083 × PI 604506) at 30 dpi ranged between 53 and 64% (Table 3), while in F_2_ (MU-CU-16 × PI 604506), the *R*^2^ percentages of QTLs linked to the same candidate region decreased from 15 dpi (*R*^2^ = 65%) to 30 dpi (*R*^2^ = 33–42%). These results suggest the requirement of other *loci* fixed in the *C. moschata* genetic background needed in the mechanism of resistance to ToLCNDV, but segregating in *C. pepo*. With this information, it is recommended to select for resistance at 30 dpi in populations coming from interspecific crosses, as it is the final stage of infection that better reflect the final response of the plants to the virus.

Resistance to ZYMV was found in different *C. moschata* accessions (Munger and Provvidenti, 1987; Paris et al., 1988; Wessel-Beaver, 2005). The resistance in the Portuguese *C. moschata* accession, Menina, is conferred by one dominant gene, Zym-1, in the cross with the susceptible *C. moschata* Waltham Butternut (Paris et al., 1988). However, when the resistance from Menina was introgressed into the *C. pepo*, segregation did not adjust to a single-gene ratio, and other additional dominant genes, Zym-2 and Zym-3, seemed to be involved in the resistance (Paris and Cohen, 2000). According to Pachner et al. (2011), even Zym-1, Zym-2, and Zym-3 together in *C. pepo* do not confer the same level of resistance seen in “Menina.” Studies of inheritance of ZYMV resistance showed that the presence of Zym-1 is essential, but must be combined with other six genes to obtain different levels of expression and durability of resistance in *C. pepo* (Pachner et al., 2015; Capuozzo et al., 2017). In accordance with these works, future QTL analysis of F_2_ (MU-CU-16 × PI 604506), including genotyping with SNPs covering the whole *Cucurbita* genome, are crucial to reveal epistatic effects of other *loci* affecting ToLCNDV resistance.

The major *locus* for resistance to ToLCNDV in chromosome 8 of both *C. moschata* sources, PI 604506 and PI 381814, is recessively inherited. Recessive resistance genes, or susceptibility genes, because their presence conditions virus susceptibility (Garcia-Ruiz, 2018), are a common defense strategy against plant viruses (Diaz-Pendón et al., 2004; Kang et al., 2005). In cucurbits, recessive resistance genes have been reported in several viruses. Translation initiation factors eIF(iso)4E and eIF4G confer recessive resistance against a subset of viruses in several crop species (Hashimoto et al., 2016). The *nsv* recessive gene, encoding an eIF4E factor, confers resistance to *melon necrotic spot virus* (Nieto et al., 2006), preventing the accumulation of viral RNA at the single-cell level (Díaz et al., 2002). In potyvirus-infected *Nicotiana benthamiana* leaf tissues, DEAD-box RNA helicase RH8, which share sequence homology with eIF4A, a component of the eIF4F multiprotein complex, is involved in viral genome translation and replication (Huang et al., 2010). We searched for putative eIF4E and eIF4F at the candidate region for ToLCNDV resistance of *C. moschata* annotation reference genome and found that two genes (CmoCh08G001290 and CmoCh08G001490) encoding an ATP-dependent RNA helicase and chromodomain-helicase-DNA-binding protein 1-like protein, respectively, mapped on the candidate region of chromosome 8. Concretely, CmoCh08G001490 is a single-copy gene in *C. moschata*, with a 3′ UTR SNP variant in PI 604506 sequence and is syntenic with a basic leucine zipper (BZIP) domain class transcription factor gene (MELO3C022278) of the chromosome 11 of *C. melo*.

In addition, other strategies have been reported for recessive resistance against viruses. The recessive *cmv1* gene that confers resistance to *cucumber mosaic virus* in melon encodes a vacuolar protein sorting 41 (VPS41) (Giner et al., 2017) involved in membrane trafficking to the vacuole. Membrane components are key factors required for plant infection success, and viral replication is associated with host intracellular membranes (Nicaise, 2014). In the case of *tom1* and *tom2A Arabidopsis* mutants, *tobacco mosaic virus* (TMV) accumulation is suppressed in single cells. Both genes encode transmembrane proteins localized in the tonoplast that are required for tobamovirus replication (Ishibashi et al., 2012). Among the annotated genes within the *C. moschata* candidate region here identified, several genes are related with membrane components. CmoCh08G001420 encodes a vesicle transport protein and CmoCh08G001500 an autophagy-related protein 3. Interestingly, two of the genes where high-impact SNPs have been detected are annotated as putative transmembrane protein (CmoCh08G001780 and CmoCh08G001790), included in the syntenic region between both candidate regions with resistance to ToLCNDV in *C. moschata* and *C. melo*.

Comparative physical mapping revealed a high level of synteny between the candidate regions with the major QTLs controlling ToLCNDV resistance of chromosomes 8 and 11 of *C. moschata* and *C. melo*, respectively (Sáez et al., 2017). The interval of ∼118 kb encompasses genes from CmoCh08G001670 to CmoCh08G001830 of *C. moschata*. Comparing the orientations of this syntenic block, the physical positions of genes in both genomes are reversed. Inversions are believed to play an important role in speciation and local adaptation by reducing recombination and protecting genomic regions from introgression (Yang L. et al., 2014). The cluster of genes within this syntenic region contains transcription factors that have been described to confer resistance to viruses in different crops.

Genes of the same family of the WRKY transcription factor-like protein of *C. moschata* (CmoCh08G001670) appears to be involved in defense responses upon TMV infection in *C. annuum* (Huh et al., 2012). In PI 604506, six 3′ UTR variants are affecting this gene. Moreover, a BZIP transcription factor gene (CmoCh08G001710) is placed close to SNP_8061105. Although CmoCh08G001470 is not placed in the syntenic region with *C. melo*, it also encodes a BZIP transcription factor gene. Particularly, a stop codon lost has been detected in this gene of PI 604506, which could alter the primary structure of the protein.

Two genes encoding MADS-box transcription factors are in this same region (CmoCh08G001750 and CmoCh08G001760). This gene family has been associated to different virus-resistance mechanisms. A MADS-box transcription factor was described as the *Ty-2* candidate, involved in the tomato resistance to *tomato yellow leaf curl virus* (TYLCV) (Yang X. et al., 2014), and recently, a MADS-box gene has been reported to be upregulated in the *Sw-7* resistance to *tomato spotted wilt tospovirus* (TSWV) (Padmanabhan et al., 2019). No SNPs with high-impact predicted effect were identified in CmoCh08G001760 between *C. moschata* accessions, but changes in 5′ and 3′ UTRs and a missense mutation with predicted moderate effect were polymorphic between resistant and susceptible accessions. This gene has no ortholog in *C. pepo* chromosome 17, likely due to the insertion affecting this region of the genome. The possible involvement of this gene in ToLCNDV resistance would explain the total susceptibility to ToLCNDV found within *C. pepo* species (Sáez et al., 2016) and the difficulties to introgress the resistance *locus* from *C. moschata* to *C. pepo.*

The CmoCh08G001760 gene has paralogs in different chromosomes of *C. moschata*. OrthoMCL detected eight putative paralogs in different chromosomes of *C. moschata* (Chr1, Chr8, Chr12, Chr14, Chr17, and Chr18). The alignment of the aa sequences of the *C. moschata* paralogs and all MADs-box genes of *Arabidopsis thaliana* shows that CmoCH08G001760.1 is most similar to the *C. moschata* paralog located in Chr17, CmoCh17G013780.1. Both genes clustered together and apart from *A. thaliana* genes (Supplementary Figure 2). The detailed comparison of the aa sequences of both genes showed significant length differences (169 aa versus 71 aa, for CmoCH08G001760 and CmoCh17G013780.1 respectively). Both proteins have a common MADs motif at the N-terminus of the protein but differ in the rest of the sequence. These results are consistent with a different function of both genes. The sequence comparison of the CmoCh17G013780.1 gene of both parentals, PI 604506 and PI 419083 (done using the genomic sequences available at NCBI under BioProject PRJNA604046), does not provide SNP variants between them, also supporting the absence of a role of this paralog in ToLNDV resistance.

Molecular markers located close to the QTLs detected here can be used in marker-assisted selection in breeding ToLCNDV-resistant pumpkins and squash. Further genetic and transcriptomic studies of the candidate genes for resistance to ToLCNDV in the different cucurbit sources of resistance analyzed to date, are needed to develop strategies to control virus useful in different species of this crop family.

## Data Availability Statement

All datasets generated for this study are included in the article/Supplementary Material.

## Author Contributions

CS, BP, and CL conceived and designed the research. CS, CL, and AS performed the tests with ToLCNDV. CS, CM, CE, and BP conducted the marker development and mapping analysis. JM-P, JB, and CS performed the bioinformatics analysis of the genomic variation and synteny. CS and BP conducted and wrote the manuscript with important contributions from JM-P, MF, and CL. All authors read and approved the final manuscript.

## Conflict of Interest

The authors declare that the research was conducted in the absence of any commercial or financial relationships that could be construed as a potential conflict of interest.

## Footnotes

1 http://cucurbitgenomics.org

2 http://cucurbitgenomics.org

3 https://github.com/JoseBlanca/

4 https://www.dnazoo.org/assemblies/Cucurbita_moschata

5 https://www.dnazoo.org/assemblies/Cucurbita_pepo

6 http://cucurbitgenomics.org

7 https://www.dnazoo.org/assemblies/

## Supplementary Material

FIGURE S1 Dot plot showing the alignment between the QTL region in the previous assembly and the new assembly. **(A)** Chromosome 8 of *C. moschata* assembly v.1 vs. scaffold 15 of the new assembly, **(B)** chromosome 17 of *C. pepo* v.4.1 and scaffold 17 of the new assembly and **(C)** syntenic region for *C. moschata* and *C. pepo* new assembly.

FIGURE S2 **(A)** Maximum likelihood tree, built using IQ-TREE v.1.5.2 (Nguyen et al., 2015), of amino acid sequences of *Arabidopsis thaliana* MADs-box and CmoCh08G001760 paralogs (in bold). Bootstrap values higher than 0.5 and lower than 0.95 are shown in the tree. Nodes with bootstrap values lower than 0.5 have been collapsed. **(B)** Conserved motifs found in all the *C. moschata* paralogs of gene CmoCH08G001760 by MEME Suite v.5.0.3. Red box represent the MAD motif.

TABLE S1 List of SNP markers polymorphic in the F2 population derived from the cross PI 419083 × PI 604506. Their positions in the *Cucurbita moschata* genome are according to the Version 1 (http://cucurbitgenomics.org/). The positions in the genetic map is according to the genetic map constructed with the F2 plants in this study and used for QTL analysis.

TABLE S2 SNPs used in all the QTLs validations. The chromosome and genetic position in each *C. moschata* × *C. moschata* and *C. pepo* × *C. moschata* linkage maps is indicated. Genomic position is shown in the last version of the *C. moschata* (v1.0), C. pepo (v4.1) and C. melo (v3.6.1) genomes available in Cucurbit Genomics Database (http://cucurbitgenomics.org). Flanking sequence of all markers is provided as well as the oligonucleotides used in the HRM genotyping assay. Markers that failed to show the expected polymorphism between parental sequences are marked as no data (−). Query cover, identity and *E*-value of Blast alignments with chromosome 11 of *C. melo* is also provide. Markers without significant similarity with chromosome 11 of *C. melo*, considering a minimum overlap between sequences of 70%, are shown as not applicable data (n/a). Striped and dotted lines are enclosing the interval position of the QTLs identified in the F2 (PI 419083 × PI 604506) and F2 (MU-CU-16 × PI 604506), respectively.

TABLE S3 List of SNPs with their predicted effect on gene function on candidate region for *C. moschata* parents and for the six transcriptomes of *C. moschata*. Gene affected, SNP location, reference and alternative alleles, allele causing the predicted high impact, type of change and genotype for each accession is shown. Note that genotypes are coded with number of allele, where 0 means the reference allele and 1,2,3 refer to the alternative allele in the displayed order. Genes with related function to plant virus resistance are marked with ^∗^.

TABLE S4 Genes annotated in the candidate region of chromosome 8 of *C. moschata*. Their paralog genes in chromosome 17 of *C. moschata* are shown, as well as BLAST alignment results with chromosome 17 of *C. pepo*. Those genes with none paralogs copies identified along the *C. moschata* genome are marked with ^∗^.

TABLE S5 Statistical significance of synteny between homologous genes in candidate region for ToLCNDV resistance in chromosome 8 of *C. moschata* and chromosome 11 of *C. melo* genomes v1.0 and v3.6.1, respectively. Genes shown in bold are indicating the pick LOD in both QTLs analysis Cucurbita populations and *C. melo*, respectively. Dotted lines are defining the candidate region of *C. melo* were the major locus, responsible of the resistance to ToLCNDV, was identified.

TABLE S6 Annotation results and fasta sequence of the Long Terminal Repeats (LTR) retrotransposon in *C. pepo* genome. The region was annotated according to the methodology for annotate repetitive elements described at Montero-Pau et al. (2018).
