# Supplementary figures and images for: A Major QTL Located in Chromosome 8 of Cucurbita moschata Is Responsible for Resistance to Tomato Leaf Curl New Delhi Virus
Source: Front Plant Sci. 2020 Mar 20;11:207. doi: 10.3389/fpls.2020.00207 (PMC7100279; doi:10.3389/fpls.2020.00207)

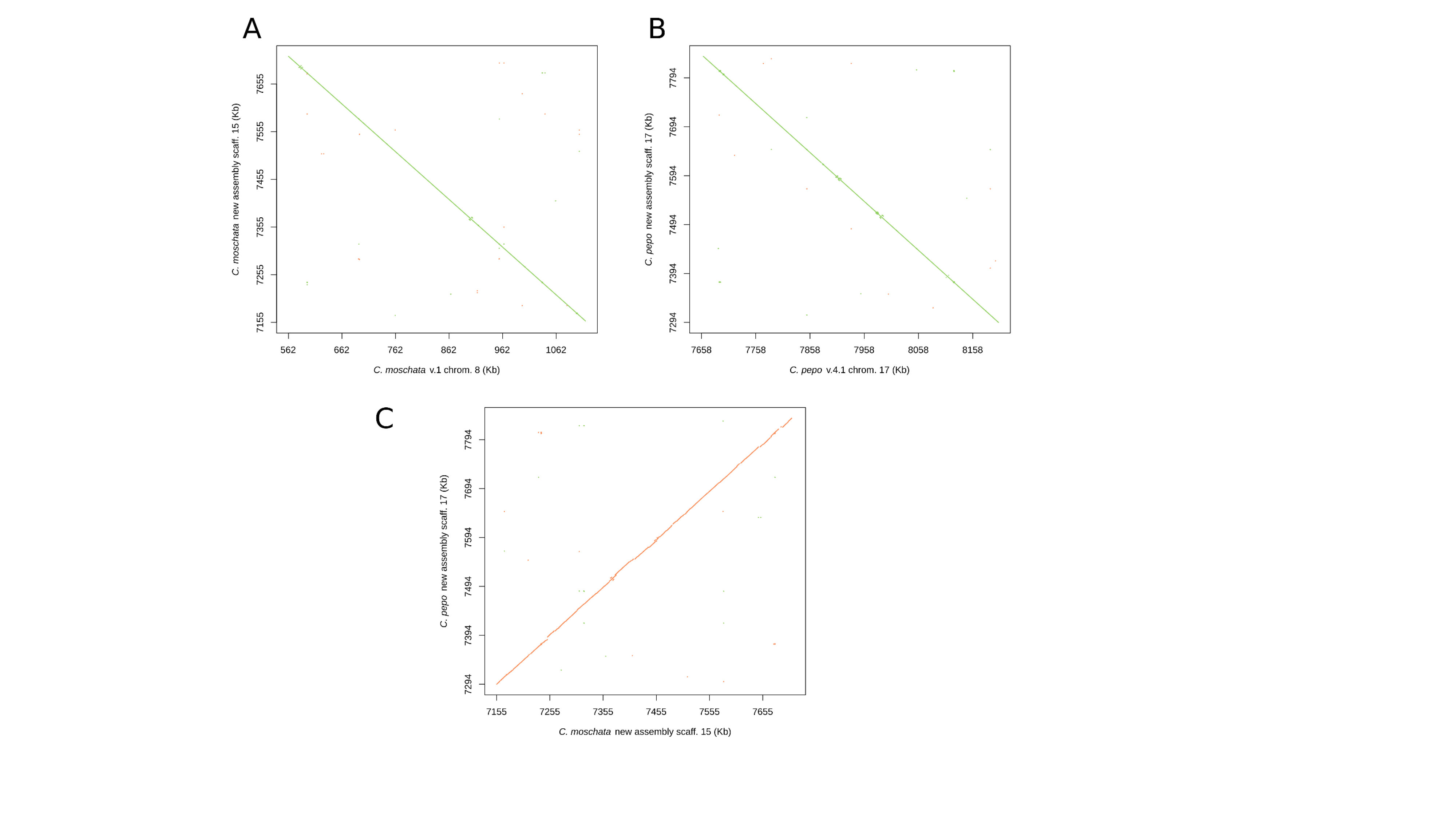

Supplement: FIGURE S1 — Dot plot showing the alignment between the QTL region in the previous assembly and the new assembly. (A) Chromosome 8 of C. moschata assembly v.1 vs. scaffold 15 of the new assembly, (B) chromosome 17 of C. pepo v.4.1 and scaffold 17 of the new assembly and (C) syntenic region for C. moschata and C. pepo new assembly. [file Image_1.tif]

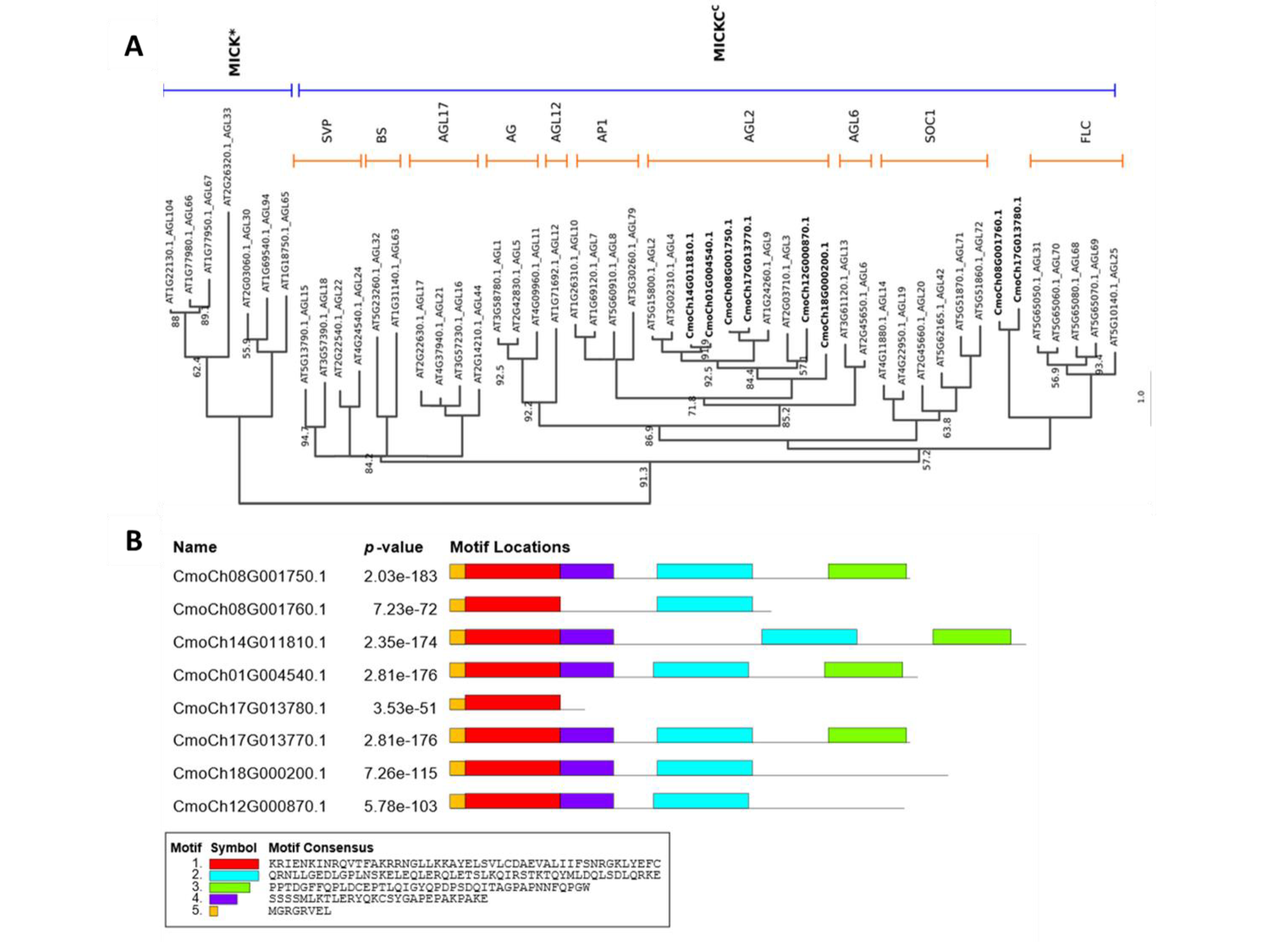

Supplement: FIGURE S2 — (A) Maximum likelihood tree, built using IQ-TREE v.1.5.2 (Nguyen et al., 2015), of amino acid sequences of Arabidopsis thaliana MADs-box and CmoCh08G001760 paralogs (in bold). Bootstrap values higher than 0.5 and lower than 0.95 are shown in the tree. Nodes with bootstrap values lower than 0.5 have been collapsed. (B) Conserved motifs found in all the C. moschata paralogs of gene CmoCH08G001760 by MEME Suite v.5.0.3. Red box represent the MAD motif. [file Image_2.tif]
